# Roles of Skeletal Muscle-Derived Exosomes in Organ Metabolic and Immunological Communication

**DOI:** 10.3389/fendo.2021.697204

**Published:** 2021-09-14

**Authors:** Wataru Aoi, Yuko Tanimura

**Affiliations:** ^1^Division of Applied Life Sciences, Graduate School of Life and Environmental Sciences, Kyoto Prefectural University, Kyoto, Japan; ^2^Department of Sport Research, Japan Institute of Sports Sciences, Tokyo, Japan

**Keywords:** skeletal muscle, myokine, exosome, metabolism, microRNA

## Abstract

Skeletal muscles secrete various factors, such as proteins/peptides, nucleotides, and metabolites, which are referred to as myokines. Many of these factors are transported into extracellular bodily fluids in a free or protein-bound form. Furthermore, several secretory factors have been shown to be wrapped up by small vesicles, particularly exosomes, secreted into circulation, and subsequently regulate recipient cells. Thus, exosome contents can be recognized as myokines. In recipient cells, proteins, microRNAs, and metabolites in exosomes can regulate the expression and activity of target proteins associated with nutrient metabolism and immune function. The levels of circulating exosomes and their contents are altered in muscle disorders and metabolic-related states, such as metabolic dysfunction, sarcopenia, and physical fitness. Therefore, such circulating factors could mediate various interactions between skeletal muscle and other organs and may be useful as biomarkers reflecting physiological and pathological states associated with muscular function. Here, this review summarizes secretory regulation of muscle-derived exosomes. Their metabolic and immunological roles and the significance of their circulating levels are also discussed.

## Introduction

In the last 20 years, skeletal muscle has emerged as a secretory organ. Early studies showed that interleukin-6 (IL-6) is a typical secretory protein produced by muscle cells (1). Thereafter, various proteins and peptides were identified as muscle-secreted factors. Classically, muscles are known to generate and secrete a metabolite, lactate through glycolysis during muscle contraction. The majority of lactate is consumed as an energy substrate in metabolic organs, a process known as the “lactate shuttle” (2). Furthermore, novel metabolites have been reported using developed metabolomic analysis techniques. Many secretory factors are transported into the circulation and mediate the communication between skeletal muscle and other organs, referred to as the myokine theory (1). In recipient cells, myokines can regulate the expression and activity of target proteins in the muscle itself in autocrine and paracrine manners or other organs *via* an endocrine route, thereby contributing to physiological and pathological phenotypes, such as metabolic capacity, muscle mass, bone density, hormone secretion, cognitive function, and tumorigenesis.

**Figure 1:**
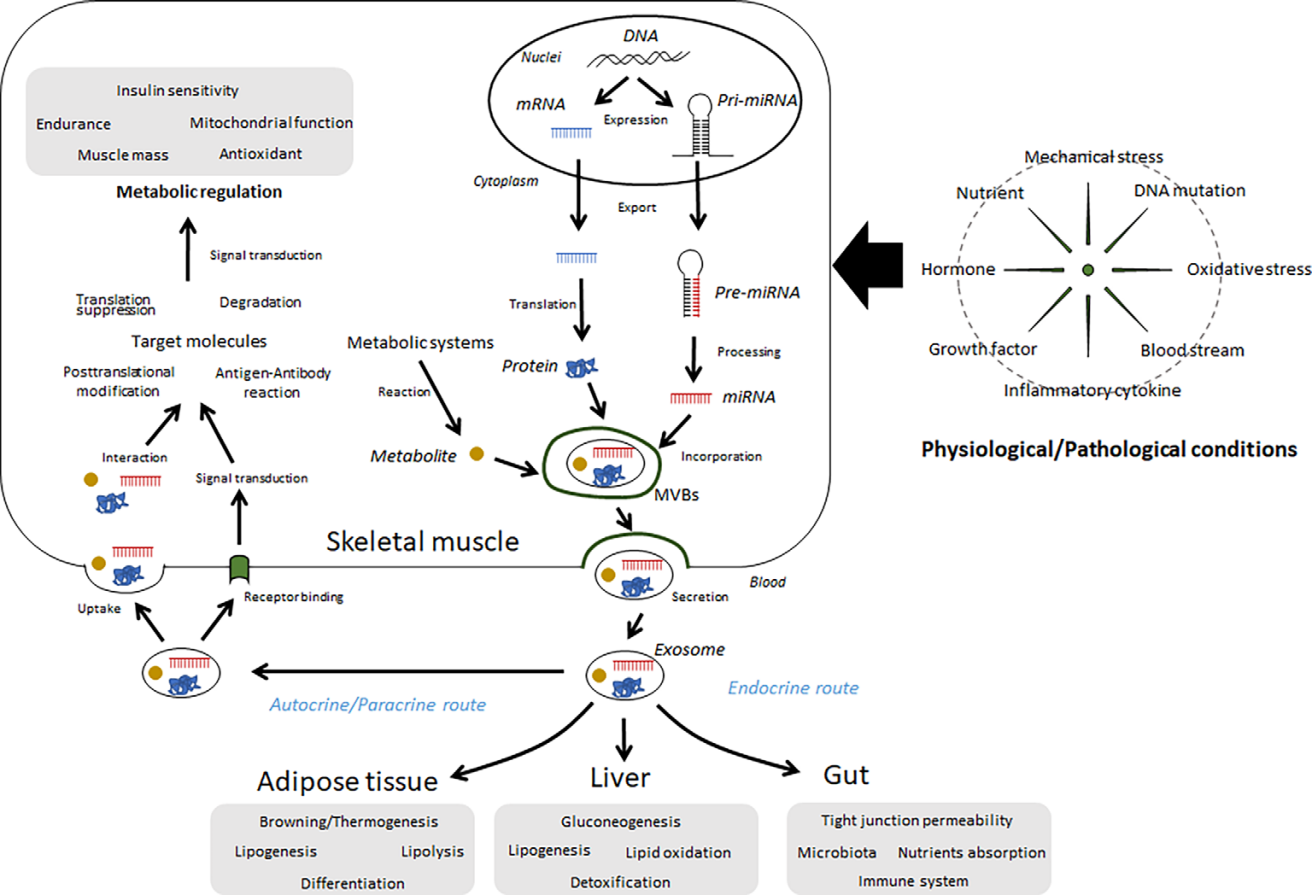
Summary of the regulation and functions of exosomes in skeletal muscle. Several miRNAs, proteins, and metabolites are wrapped up by intracellular vesicles, such as exosomes, in skeletal muscle cells and secreted into extracellular fluids. These components can be affected by various conditions, including physical activity, diet, diseases, and aging. Circulating exosomes migrate to the skeletal muscle as well as other organs and regulate metabolic and immunological functions.

Many secretory factors exist in free form or in a protein-bound form in circulation. Growing evidence shows that several secreted factors also contain in extracellular vesicles (EVs). Generally, two major subtypes of EVs have been characterized with differences of their biogenesis. In contrast to plasma membrane-derived “microvesicles”, “exosomes” are defined as the EVs derived from endosome (3). Although it is difficult to distinguish each subtype completely, EVs differ based their components and functions. A noncoding RNA, microRNA (miRNA) is a typical component. Over 60% of protein-coding genes may be regulated by miRNAs (4), and many of these miRNAs are thought to play important roles in a range of biological processes. Some intracellular miRNAs are wrapped up by small vesicles, particularly exosomes, secreted into circulation, and then regulate recipient cells. Indeed, over 1000 RNAs have been detected in exosomes (5), in addition to proteins/peptides, lipids, and metabolites. Secreted exosomes dock to the plasma membrane of target cells, where they can bind or fuse with the plasma membrane or be endocytosed and then deliver their cargo (6). Through this process, exosomes mediate cell-cell and organ-organ communication (**Figure 1**). Skeletal muscle cells secrete exosomes; thus, their contents can be recognized as myokines and have potential functions over free-form secretory factors.

Here, we discuss the significance of circulating exosomes and their contents in the context of various physiological, and pathological states, such as muscle disorders, lifestyle-related diseases, and physical fitness.

## Exosome Components and Functions Secreted From Skeletal Muscle

The production of EVs, which includes exosomes, by skeletal muscle cells was first demonstrated by Guescini et al. (7). In a proteomic assay, muscle-derived exosomes were found to contain exosome-associated proteins and signal transduction proteins. Interestingly, mitochondrial DNA was also shown to be taken up into exosomes and released into the extracellular medium in cultures of C2C12 cells (7). In parallel, miRNAs have been reported to be present in blood. Several miRNAs are highly enriched in muscle tissues and are often referred to as myomiRs. Four myomiRs, namely, *miR-1*, *miR-133a*, *miR-133b*, and *miR-206*, together account for nearly 25% of miRNA expression in skeletal muscles in both humans and mice (8, 9) and contribute to the development of animal and human skeletal muscle cells (10, 11). Skeletal muscle cells were shown to secrete exosomes containing these four myomiRs (12, 13), which were found to be involved in myoblast differentiation into myotubes *via* an autocrine route. In addition, several animal and human studies have suggested the existence of some circulating myomiRs in the living body (14–16). Recently, intact skeletal muscle tissues secrete exosomes containing *miR-1*, *miR-133a*, *miR-133b*, and *miR-206*, which depends on their expression levels in the tissues (13). In addition to the four miRNAs, several muscle-enriched miRNAs, including *miR-208*, *miR-486*, and *miR-499*, have also been identified in circulation (14, 17). Additionally, in human satellite cells, extracellular guanosine 5’-triphosphate was shown to increase the level of myomiRs and also induce the release of exosomes containing guanosine (18). These studies suggest that circulating muscle-specific miRNAs likely can act as myogenetic factors in autocrine and paracrine manners, and some are even released into circulation (13). However, their involvement in sarcopenia and training-induced muscle hypertrophy is unclear.

Although few proteins have been identified in exosomes secreted from skeletal muscle, several proteins related to the growth and metabolism of muscle have been reported. Proteomic findings using two-dimensional gel electrophoresis and liquid chromatography tandem mass spectrometry have shown that exosomes secreted from C2C12 cells show different in compositions after differentiation into myoblasts and myotubes. Several protein components, including integrin subunit beta 1, CD9, CD81, neural cell adhesion molecule, CD44, and myoferlin, likely play key roles in the differentiation of these cells (19). Additionally, growth factors such as insulin-like growth factors and fibroblast growth factor-2 are also present in exosomes from differentiating human skeletal muscle cells (20). Such exosomes likely contribute to skeletal myogenesis. A previous study showed that mouse and human myoblasts cultured in exosome-depleted serum form fewer myotubes than myoblasts grown in normal serum (21). This effect was not reversed, even when the medium was replaced with normal medium. Moreover, the expression levels of myomiRs, such as *miR-1*, *miR-206*, and *miR-133a*, are decreased during myoblast proliferation. As mentioned above, exosomes containing guanosine may also contribute to regulated contents of myomiRs (18). In addition, differentiating C2C12 exosomes increase motor neuron survival in NSC-34 cells (22). Therefore, skeletal muscle-secreted exosomes can mediate the maintenance of physiological homeostasis in muscle tissue in autocrine and endocrine manners.

In general, there are two pathways for exosome formation: endosomal sorting complex required for transport (ESCRT)-dependent and ESCRT-independent pathways (23). ESCRT-dependent pathways involve four types of ESCRT complexes and the proteins that bind to them which regulate protein recruitment, vesicle budding and separation. On the other hand, the ESCRT-independent pathway is mediated by sphingolipid ceramide *via* neutral sphingomyelinase. Although the regulatory mechanism of their biogenesis and secretion in muscle have not been fully clarified, several studies have provided some information. Using proteomic and bioinformatic analysis, Forterre et al. (12) suggested the existence of ESCRT proteins in exosomes from C2C12 differentiation. The release of EVs from C2C12 myoblasts was found to be modulated by intracellular ceramide levels in a Ca^2+^-dependent manner (15). In *mdx* mice, the inhibition of ceramide synthesis suppressed EV secretion and improved muscle dystrophy (15).

Since the report by Valadi et al. (5), evidences regarding not only the secretory mechanism but also body dynamism of exosomes have been developed. Particularly, Aswad et al. (24) showed that information. fluorescently labeled muscle-derived exosomes injected into mice were found to be taken up into various tissues, including the lung, liver, spleen, brain, heart, pancreas, and gastrointestinal tract. In addition, a high-fat diet was shown to accelerate the release of exosomes from muscle tissues in mice (24). Muscle-derived exosomes from mice fed a high-fat diet induced myoblast proliferation and altered the expression of cell cycle and differentiation factors. These findings suggest that muscle-derived EVs have paracrine signaling functions *via* alteration of muscle homeostasis in response to a high-fat diet and may have endocrine functions *via* targeting other tissues *in vivo*. The same group showed that EVs from the muscles of mice on a high-fat diet increased the sizes of isolated islets *in vitro* and induced the proliferation of murine insulin-secreting MIN6B1 cells (25).

## Exosomes in Physical Activity/Muscle Contraction

Skeletal muscles function as a supporting organ during physical activity. The muscles consume lipids and glucose as substrates and generate energy for muscle contraction; thus, exercise training adaptively leads to muscle building and endurance. In addition, daily physical activity reduces the risk of various noncommunicable diseases, such as cardiovascular disease, type 2 diabetes, and cancer. Habitual exercise also counteracts the development of age-related muscle atrophy by regulating myogenesis and protein metabolism. Muscle-secreted factors have been known to contribute to physical fitness, preventive diseases, and anti-aging effects.

Initial studies have shown that circulating miRNAs are altered in response to a single bout of exercise and training (14, 26–30). However, the miRNAs are not limited to the cargo in exosomes and their secretory sources cannot be identified. In a last decade, EVs have been isolated in circulation and their secretory characteristics examined in physiological and pathological states (**Table 1**). Differences in exosomal or vesicle-free contents may affect the results. For example, Guescini et al. (31) reported that muscle-enriched *miR-133b* and *miR-181a-5p* in EVs were elevated after acute exercise, and a positive correlation was found between aerobic fitness and circulating muscle-enriched miRNAs. Additionally, Yin et al. (17) examined the secretory dynamics of cell-free and exosomal muscle-enriched miRNAs with exercise patterns in rats. In cell-free miRNAs, only *miR-1* and *miR-133a* were increased immediately after uphill exercise, whereas more miRNAs (*miR-1*, *miR-133a*, *miR-133b*, *miR-206*, *miR-208a*, and *miR-499*) increased after downhill running. In exosomes, only *miR-133a* increased following uphill running, whereas *miR-1*, *miR-133a*, and *miR-499* increased after downhill running (17). Mild to moderate muscle-damaging exercise affects *miR-31* in circulating EVs after exercise but does not alter myomiRs in EVs (32). Although the levels of exosomal miRNAs do not change as easily in response to exercise, training status can influence the response. For example, Nair et al. (33) showed that regular exercise adaptively increased baseline levels of *miR-486-5p*, *miR-215-5p*, and *miR-941* and decreased the levels of *miR-151b*. These miRNAs were associated with IGF-1 signaling. In particular, circulating *miR-486*, a muscle-enriched miRNA, is altered in response to exercise and correlated with endurance capacity (14), suggesting that it may act on certain recipient cells. A major putative target of *miR-486* is phosphatase and tensin homolog (PTEN), which is a negative regulator of insulin signal transduction. Therefore, exosomal *miR-486* has a potential for protein synthesis through the IGF-1/Akt/mTOR pathway. Release of EVs into circulation in response to exercise is also affected by the physical characteristics of the individuals, including sex, body mass index, and fitness level (44). Furthermore, as reported by Whitham et al. (45), many proteins are contained in exosomes and small vesicles, and their profiles can be changed by exercise. Therefore, future investigations should focus on proteins and peptides as cargo in exosomes.

**Table 1:** Muscle-derived exosome components in physiological and pathological states.

Status			Subject	Change	Components	Reference number
Exercise	Acute	• Cycling	Healthy young (serum)	↓	miR-486	(14)
		• Running	Healthy young (EVs)	↑	miR133b, miR-181a-5p	(31)
		• Uphill running	Rat (cell-free)	↑	miR-1, miR-133a	(17)
		• Uphill running	Rat (exosome)	↑	miR-133a	(17)
		• Downhill running	Rat (cell-free)	↑	miR-1, miR-133a, miR-133b, miR-206, miR-208a, miR-499	(17)
		• Downhill running	Rat (exosome)	↑	miR-1, miR-133a, miR-499	(17)
		• Jump and downhill running	Healthy young (plasma)	↓	miR-31	(32)
				→	myomiRs	
	Chronic	• Cycling	Healthy young (serum)	↓	miR-486	(14)
		• Running, cycling	Healthy elderly (exosome)	↑	miR-486-5p, miR-215-5p, miR-941	(33)
				↓	miR-151b	
Disease	Neuromuscular disorder	• Duchenne muscular dystrophy	Mouse (serum)	↑	miR-1, miR-133a, miR-206	(34)
		• Duchenne muscular dystrophy	Patient (serum)	↑	myomiRs	(35)
		• Denervation	Mouse (exosome)	↑	miR-206	(16)
				↓	miR-1, miR133a, miR 133b	
	Diabetes	• Streptozotocin treatment	Rat (skeletal muscle)	↓	miR-23a	(36)
		• Dexametazon treatment	Cultured myotube (exosome)	↑	miR-23a	
		• Type 2 diabetes	Rat (serum)	↑	miR-144	(37)
			Patient (serum)	↑	miR-144	
		• Precursor miR-23a/27a injection	Mouse (skeletal muscle)	↑	miR-23a, miR27a	(38)
	Chronic kidney disease	• Exo/miR-26a injection	Mouse (skeletal muscle)	↑	miR-26	(39)
	Chronic obstructive lmonary disease		Sarcopenic condition (serum)	↑	miR-1, miR-206, miR-499	(40)
	Rhabdomyosarcoma tumors		Patient (serum)	↑	miR-1, miR-133a, miR-133b, miR-206	(41)
Aging		• Aging	Mouse (serum)	↑	miR-29b, miR-34a	(42)
Diet	High fat diet	• Obesity	Mouse (sketetal muscle)	↑	exosome	(24)
Ischemia reperfusion			Mouse (sketetal muscle)	↑	complement component C3 prepropeptide, PK-120 precursor, alpha-amylase 1 precursor, beta-enolase isoform 1, adenylosuccinate synthetase isozyme 1	(43)
	
	

↑, increase; ↓, decrease; →, no change.

## Muscle-Derived Exosomes and Noncommunicable Diseases

miRNAs have been shown to function as modulators of myogenesis, muscle mass, and metabolic capacity in skeletal muscle (46–48). In addition, several muscle-enriched miRNAs are released into the circulation, and these miRNAs can be altered by muscle disorders (**Table 1**). Therefore, several researchers have investigated the functions of these miRNAs in the muscle itself and in other organs through paracrine and endocrine routes. In animal models and human patients of Duchenne muscular dystrophy, circulating levels of *miR-1*, *miR-133a*, and *miR-206* were suggested to be increased and were correlated with the progression of muscle pathology (34, 35). Another neuromuscular disorder, denervation, resulted in higher levels of *miR-206* and lower levels of *miR-1*, *miR-133a*, and *miR-133b* in muscle-derived exosomes (16). These secretory characteristics of miRNAs may be closely involved in the development of pathological muscle conditions. Indeed, exposure of C2C12 muscle cells to EVs from the serum of *mdx* mice resulted in a decrease in cell death (15). Moreover, inhibition of ceramide synthesis and injection with GW4869, an inhibitor of exosome secretion, ameliorate muscular dystrophy in *mdx* mice, suggesting that EVs and myomiRs are closely associated with skeletal muscle degeneration.

Several exosomal contents have been shown to change with associated metabolic dysfunction. For example, *miR-23a* levels were found to be decreased in atrophied gastrocnemius muscles from streptozotocin-induced diabetic rats. Additionally, *miR-23a* was present in exosomes isolated from the medium of C2C12 myotubes, and dexamethasone treatment increased its abundance in exosomes (36). Thus, selective packaging of *miR-23a* into exosomes may reduce its content in muscles. Jalabert et al. (25) found that skeletal muscle-derived exosomes injected into mice could specifically target *miR* pancreatic islet cells and affect gene expression and proliferation of βcells. Although direct evidence has not yet shown whether muscle-derived exosomes can influence insulin secretion, regular exercise is known to promote the secretory capacity of insulin (49, 50). Karolina et al. (37) found that circulating *miR-144* levels are increased in animals and humans with type 2 diabetes. This elevation is negatively correlated with insulin receptor substrate 1 in insulin-responsive tissues, including skeletal muscle, suggesting that the elevation of *miR-144* levels in circulation may be associated with the development of insulin resistance. Furthermore, overexpression of *miR-23a/-27a* in muscle prevents diabetes-induced muscle cachexia and attenuates renal fibrosis lesions *via* muscle-kidney crosstalk (38). In addition, overexpression of *miR-26* in muscle prevents chronic kidney disease (CKD)-induced muscle wasting *via* exosomal *miR-26a* (39). This result supports the potential therapeutic applications of exosome delivery of specific miRNAs to treat and prevent CKD and sarcopenia.

Levels of muscle-specific miRNAs, i.e., *miR-1*, *miR-206*, and *miR-499*, were also higher in the plasma of patients with chronic obstructive pulmonary disease (COPD), who often have a sarcopenic condition (40). In plasma samples from 103 patients with COPD and healthy controls, plasma *miR-499* and *miR-206* levels were associated with inflammatory responses in skeletal muscle and circulation (40). This may cause sarcopenic conditions in patients with COPD. In addition, serum levels of myomiRs (i.e., *miR-1*, *miR-133a*, *miR-133b*, and *miR-206*) were significantly higher in patients with rhabdomyosarcoma tumors than in those with non-rhabdomyosarcoma tumors (41). Ischemia-reperfusion stimulation of skeletal muscle induces different responses of circulating exosomal proteins between nuclear factor-κB-knockout and wild-type mice (43). The results showed that various proteins, including complement component C3 prepropeptide, PK-120 precursor, alpha-amylase one precursor, beta-enolase isoform 1, adenylosuccinate synthetase isozyme 1, and glyceraldehyde-3-phosphate dehydrogenase, were altered. Interestingly, exosome-like vesicles released from inflamed myotubes induce myoblast inflammation and inhibit myogenic mechanisms while stimulating atrophic signals (51), supporting the roles of muscle-derived EVs in the development of inflammatory diseases *via* organ-organ crosstalk.

Several studies have shown that aging and dietary habits can also affect the quality of muscle-derived exosomes. An aging-dependent miRNA, *miR-29b-3p*, was observed in exosomes isolated from differentiated atrophic myotubes. The *miR-29b-3p*-containing exosomes released from myotubes were taken up by neuronal SH-SY5Y cells, leading to downregulation of neuronal-related genes and inhibition of neuronal differentiation. Muscle-derived *miR-34a* also increases with age in circulating EVs and induces the senescence of bone marrow stem cells (42). Thus, such age-associated exosomes in circulation may promote aging through modulation of crosstalk between muscle cells and neurons. In addition, a high-fat diet can promote the secretion of exosomes from skeletal muscles, thereby affecting proliferation and differentiation of myoblast (24). Thus, exosomes contribute to lipid transfer through mediating communication between cells in muscle tissues. Regarding the cardiovascular system, the muscle-derived exosomes with angiogenic miRNAs such as *miR-130a*, induces angiogenesis *via* redox signaling in endothelial cells (52). This prevents age-related loss of capillarization, found in type 2 diabetes and chronic heart failure. Furthermore, the influence on the function and survival of β-cells under normal and diabetic conditions has been discussed (25). *miR-133a*, *miR-206*, and *miR-16* in muscle-derived exosomes likely result in the abnormal regulation of insulin secretion in pancreatic islets (53). In contrast, *miR-133a*, *miR-133b*, and *miR-206* may have a tumor suppressive function. Under several cancer conditions, the levels of these myomiRs levels in circulation are lower than in the normal conditions (54). Although the relationship between exosomes and disease is still unclear, these observations support the significant role of muscle-derived exosomes in physiological homeostasis and pathological changes.

## Conclusion

In this review, we discussed the roles of exosomes in metabolic and immunological communication between muscles and other organs. Many components are contained in muscle-derived exosomes and are likely contributed to maintaining or impairing the homeostasis of muscles and organs throughout the body. Abnormalities in glucose tolerance and lipid metabolism occur as a result of impaired metabolic function of skeletal muscle. Furthermore, it is now known that age-related muscle atrophy, or sarcopenia, is associated with central diseases including respiratory and cardiovascular diseases, chronic kidney disease, dementia, and depression. There is also a negative correlation between physical activity and the risk of developing cancer, as well as an association between intestinal bacteria and leaky gut syndrome. Exosomes may be involved in these associations between skeletal muscle and multiple organs. Thus far, the search for bioactive factors in circulation has targeted free proteins, peptides, and metabolites, but in the future it is necessary to include the components encapsulated in exosomes. In addition, exosomes have potential applications as biomarkers reflecting metabolic and immunological conditions. However, it is unclear whether muscle-derived exosomes can be useful as a biomarker to reflect physical fitness, diseases, or aging. Many factors lack specificity because common factors are secreted by multiple organs and different stimuli. Therefore, special factors with highly expressed in skeletal muscle should be found in a future. In contrast to the identification of many miRNAs in exosomes, few proteins and metabolites have been identified, although it is assumed that many exist. Accordingly, further studies are needed to elucidate the detailed characteristics of exosomes under physiological and pathological conditions.

## Author Contributions

WA and YT wrote the manuscript. All authors contributed to the article and approved the submitted version.

## Funding

This work was supported by Grants-in-Aid for Scientific Research (B) KAKENHI Grant Number JP20H04080 (WA) from Japan Society for the Promotion of Science and the grant from the Uehara Memorial Foundation.

## Conflict of Interest

The authors declare that the research was conducted in the absence of any commercial or financial relationships that could be construed as a potential conflict of interest.

## Publisher’s Note

All claims expressed in this article are solely those of the authors and do not necessarily represent those of their affiliated organizations, or those of the publisher, the editors and the reviewers. Any product that may be evaluated in this article, or claim that may be made by its manufacturer, is not guaranteed or endorsed by the publisher.
